# Phosphorus *K*-edge XANES spectroscopy of mineral standards

**DOI:** 10.1107/S0909049510045322

**Published:** 2010-12-02

**Authors:** Ellery D. Ingall, Jay A. Brandes, Julia M. Diaz, Martin D. de Jonge, David Paterson, Ian McNulty, W. Crawford Elliott, Paul Northrup

**Affiliations:** a School of Earth and Atmospheric Sciences, Georgia Institute of Technology, Atlanta, GA 30332-0340, USA; b Skidaway Institute of Oceanography, 10 Ocean Science Circle, Savannah, Georgia 31411, USA; c Australian Synchrotron, 800 Blackburn Road, Clayton, Victoria 3168, Australia; d Advanced Photon Source, Argonne National Laboratory, Argonne, IL 60439, USA; e Department of Geosciences, Georgia State University, Atlanta, GA 30302-4105, USA; f Department of Geosciences, Stony Brook University, Stony Brook, NY 11794, USA

**Keywords:** XANES, phosphorus, phosphate minerals

## Abstract

Phosphorus *K*-edge X-ray absorption near-edge structure (XANES) spectroscopy was performed on phosphate mineral specimens including (*a*) twelve specimens from the apatite group covering a range of compositional variation and crystallinity; (*b*) six non-apatite calcium-rich phosphate minerals; (*c*) 15 aluminium-rich phosphate minerals; (*d*) ten phosphate minerals rich in either reduced iron or manganese; (*e*) four phosphate minerals rich in either oxidized iron or manganese; (*f*) eight phosphate minerals rich in either magnesium, copper, lead, zinc or rare-earth elements; and (*g*) four uranium phosphate minerals. The identity of all minerals examined in this study was independently confirmed using X-ray powder diffraction. Minerals were distinguished using XANES spectra with a combination of pre-edge features, edge position, peak shapes and post-edge features. Shared spectral features were observed in minerals with compositions dominated by the same specific cation. Analyses of apatite-group minerals indicate that XANES spectral patterns are not strongly affected by variations in composition and crystallinity typical of natural mineral specimens.

## Abstract 2

Phosphorus *K*-edge XANES spectra are presented for a diverse set of 44 phosphate minerals.

## 1. Introduction

Phosphorus is a necessary component of molecules vital for energy storage and transfer, reproduction and structure in all organisms. Owing to this vital role, phosphorus availability exerts a strong control over the growth of all organisms. In vast areas of the ocean the growth of photosynthetic organisms can be limited by the availability of phosphorus (Benitez-Nelson, 2000 ▶; Karl & Björkman, 2002 ▶). The growth of photosynthetic organisms in the ocean and on land consumes large quantities of atmospheric carbon dioxide, which links phosphorus to the cycle of this environmentally important greenhouse gas. In soils, phosphorus availability is one of several factors controlling plant health and crop yields (Hinsinger *et al.*, 2001 ▶; Lombi *et al.*, 2006 ▶). In some coastal and lacustrine settings excessive phosphorus inputs from both natural and anthropogenic sources can lead to a variety of water quality problems, especially eutrophication (Sharpley & Tunney, 2000 ▶) and damage to fisheries. Phosphorus is also an important constituent in many bioremediation technologies. For example, in aquifers contaminated with uranium, precipitation of phosphate minerals has been proposed as a mechanism for uranium sequestration (Beazley *et al.*, 2009 ▶). Incorporation of phosphorus into solid phases including minerals is a key process in many sewage treatment and mariculture systems (Van Rijn *et al.*, 2006 ▶; Krom *et al.*, 1995 ▶; Valsami-Jones, 2001 ▶). Given the importance of phosphorus, the mechanisms by which it is supplied and removed from natural and artificial systems has been the focus of intense study (*e.g.* Clark *et al.*, 1998 ▶, 1999 ▶; Diaz *et al.*, 2008 ▶; Diaz & Ingall, 2010 ▶; Kolowith *et al.*, 2001 ▶; Sannigrahi & Ingall, 2005 ▶, 2006 ▶; Froelich *et al.*, 1982 ▶; Sundby *et al.*, 1992 ▶; Ingall & Jahnke, 1997 ▶; Ingall *et al.*, 2005 ▶; Young & Ingall, 2010 ▶). A key to understanding phosphorus supply, removal and transformations in all systems is knowledge of its chemical speciation.

Phosphate-containing mineral phases are common constituents of the phosphorus pool in all systems. Depending on environmental conditions, these minerals may supply biologically available phosphorus through dissolution, or sequester it *via* precipitation. Uncertainty regarding the mineral forms of phosphorus present in a system often prevails because of the difficulty associated with analyzing these phases with conventional techniques. Typically, these minerals are present in low concentrations, which often precludes direct characterization by simple chemical, optical or bulk spectroscopic methods. Microbial mediation of many processes involving phosphorus often results in the production of submicrometer-sized particles, which also creates difficulties for their characterization by conventional techniques (Brandes *et al.*, 2007 ▶; Diaz *et al.*, 2008 ▶).

Recent studies have demonstrated that phosphorus XANES provides an excellent way to examine phosphorus mineralogy and transformations in natural and agricultural systems (Diaz *et al.*, 2008 ▶, 2009 ▶; Brandes *et al.*, 2007 ▶; Seiter *et al.*, 2008 ▶; Beauchemin *et al.*, 2003 ▶; Peak *et al.*, 2002 ▶; Ajiboye *et al.*, 2007 ▶; Hesterberg *et al.*, 1999 ▶; Myneni, 2002 ▶; Sato *et al.*, 2005 ▶; Eveborn *et al.*, 2009 ▶; Kruse & Leinweber, 2008 ▶). In many of the above studies, phosphorus XANES information for phosphorus minerals is presented for only a limited number of mineral species relevant to the particular study. Other studies have presented mineral and reference compounds for the phosphorus *L*_2,3_-edge (Kruse *et al.*, 2009 ▶). As a resource for future studies utilizing phosphorus *K*-edge XANES, spectra are presented for a diverse set of 44 phosphate minerals.

## 2. Experimental

The identity, purity and crystallinity of all minerals investigated in this study were independently verified through bulk X-ray powder diffraction techniques. XANES data for all minerals are provided in the supplementary online material.^1^ All natural mineral specimens were purchased from mineral dealers, and one specimen (monetite) was obtained from a chemical supply company. Minerals were identified using powder X-ray diffraction. A Philips model 12045 diffractometer with a Bragg Brentano goniometer was used to scan mounts of approximately 1 g of finely ground phosphate mineral. The powders were scanned using Cu radiation filtered with a graphite monochromator. MDI Databox was used to provide computer control for scanning these powders.  The minerals were identified using the *JADE* program in MDI Databox, which contains International Centre for Diffraction Data for the minerals. The carbonate content of apatite-group minerals was measured with a COSTECH elemental analyzer using methods described by Hedges & Stern (1984 ▶).

XANES spectroscopy was conducted at the X15B beamline at Brookhaven National Laboratory’s National Synchrotron Light Source. X15B is optimized for light-element X-ray spectroscopy in the 1–5 keV range. Mineral spectra were collected using a monochromator with silicon (111) crystals and a flux of approximately 10^12^ photons s^−1^ focused to a 1 mm × 1 mm spot by a toroidal mirror. The monochromator was operated at full tune, with higher harmonics absorbed by an upstream vertically collimating mirror. Samples were finely ground (to minimize self-absorption), thinly mounted in 5 µm polypropylene film envelopes, and placed into a helium-purged sample chamber. Phosphorus *K* fluorescence was measured using a solid-state germanium detector positioned at 90° to the incident beam. The fluorescence signal was normalized to the incident beam intensity as monitored by an ionization chamber just before the sample. The monochromator energy was calibrated with a red phosphorus standard (Jürgensen, 2005 ▶) and subsequent experimental runs calibrated to a fluorapatite secondary standard. For phosphorus, peak center positions are less problematic to determine than edge positions, as many minerals contain pre-edge features, and edge positions (*e.g.* first derivative) may be affected by self-absorption or other experimental effects.

In parallel to these studies, micro-XANES data were collected on a subset of these mineral P standards using an X-ray microfocus beamline (2-ID-B at the Advanced Photon Source). However, drifts in the monochromator energy reproducibility and the inherent challenges of using a chromatic focusing optic (Diaz *et al.*, 2008 ▶; see also supplementary material) made this data difficult to accurately quantify. In response to these issues an in-line X-ray beam energy and intensity monitor was developed (de Jonge *et al.*, 2010 ▶) that allowed correction of the monochromator drift enabling clear detection of small changes to the location of XANES features. Simultaneous monitoring of beam energy was not required at X15B, where energy reproducibility was determined to be within 0.05 eV over repeated scans.

## 3. Results and discussion

The XANES spectra of all phosphorus minerals in this study had principal *K*-edge peak energies between 2152.9 and 2154.1 eV (Brandes *et al.*, 2007 ▶). This peak results from the excitation of an electron from a 1*s* inner orbital to a higher-energy 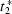 orbital as a result of interaction with an X-ray. Subsequent decay of higher-energy electrons to unoccupied 1*s* orbitals releases photons, which are counted by the germanium detector. All phosphorus minerals measured in this study contained phosphorus in the +V oxidation state. Although all *K*-edge peaks were found within the above energy range, systematic differences between mineral groups were found. Apatite minerals showed the lowest peak energies (2153.0 ± 0.1 eV). For minerals in which elements other than calcium were prevalent the *K*-edge peak energy increased, with the highest energies seen for Al (2153.6 ± 0.2 eV), U (2153.8 ± 0.1 eV) and oxidized metal (2153.9 ± 0.2 eV) phosphates. Spectra of most minerals also revealed additional pre-edge or post-edge features that can be used to identify specific minerals or differentiate between mineral groups. In general these pre- and post-edge features are related to (i) the presence and weight percent of different elements; (ii) the oxidation state of these elements; and (iii) the arrangement of these elements in the mineral structure. More information on pre-edge and post-edge features for specific minerals and mineral groups follows.

### 3.1. Apatite-group minerals

**Figure 1:**
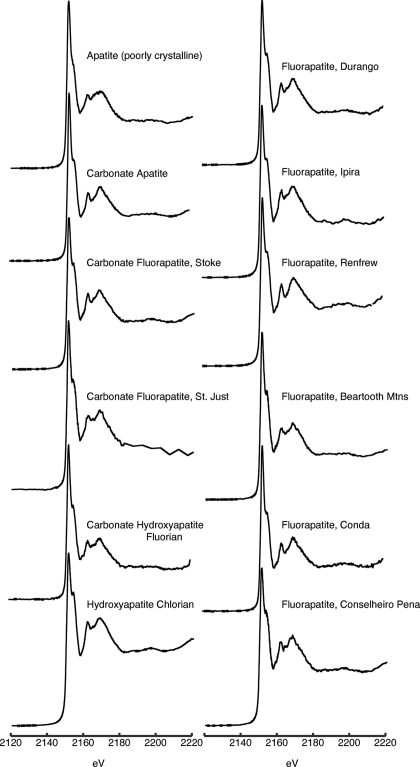
Phosphorus XANES spectra for apatite-group minerals listed in Table 1 ▶.

Information and spectra for apatite-group minerals are presented in Table 1 ▶ and Fig. 1 ▶, respectively. Apatite is a common phosphate-bearing mineral, found in igneous, sedimentary and metamorphic rocks (Deer *et al.*, 1992 ▶; Chang *et al.*, 1996 ▶). Precipitation of apatite is a significant removal mechanism for phosphorus in various natural and anthropogenic systems. Given the variety of conditions under which apatite precipitation occurs, there is typically a range in apatite composition found in natural systems that deviates from the idealized chemical formula Ca_5_(PO_4_)_3_(OH,F,Cl). In the apatite structure the hydroxyl, fluorine and chlorine atoms can substitute for each other. Fluorine- and hydroxyl-rich varieties are the most common forms found in natural systems. In addition to the fluorine and hydroxyl substitutions, calcium may be partially replaced by other elements such as manganese, strontium and rare-earth elements. These substitutions typically replace no more than a few percent of the calcium but exceptions have been observed in unusual environments (Deer *et al.*, 1992 ▶; Chang *et al.*, 1996 ▶). Carbonate anions are often incorporated in apatite minerals especially in marine environments where they form authigenically. In rare cases partial substitution of arsenate for phosphate has also been observed (White & Dong, 2003 ▶; Deer *et al.*, 1992 ▶; Chang *et al.*, 1996 ▶).

To determine the effect of compositional variations on the phosphorus XANES spectra of apatite-group minerals, a variety of samples were analyzed. These compositional variations are reflected in the names of the apatite minerals and associated idealized chemical formulas (Table 1 ▶) as determined by bulk powder X-ray diffraction analyses. Direct compositional analysis by CHN analyzer reveals that carbonate-containing apatite minerals analyzed in this study contain 0.08 to 0.81 wt% carbonate.

Despite the compositional differences of the apatite-group minerals studied, key features of the XANES spectra are similar. For apatite-group minerals, identifying features include a distinctive shoulder or widening on the main absorption edge peak centered at approximately 2155.6 eV, and higher-energy secondary peaks at 2163.3 and 2170 eV (Fig. 1 ▶). The position of spectral features observed here is consistent with those observed in other studies of apatite minerals (Peak *et al.*, 2002 ▶; Hesterberg *et al.*, 1999 ▶; Beauchemin *et al.*, 2003 ▶; Franke & Hormes, 1995 ▶; Brandes *et al.*, 2007 ▶). One apatite sample from Mono Lake, CA, USA (Table 1 ▶), clearly had lower crystallinity as revealed by broad peaks in the bulk powder X-ray diffraction patterns. This ‘poorly crystalline’ sample also exhibited less distinctive secondary peaks in its XANES spectrum (Fig. 1 ▶).

### 3.2. Non-apatite calcium phosphate minerals

**Figure 2:**
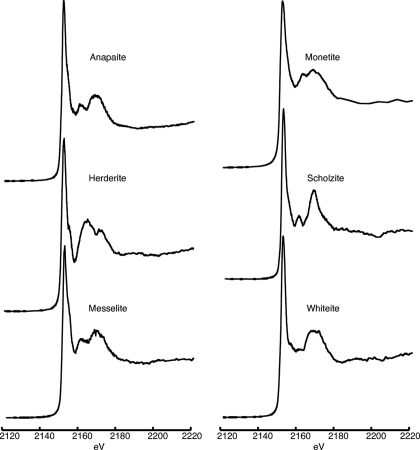
Phosphorus XANES spectra for calcium phosphate minerals (Table 2 ▶) other than apatite-group minerals.

Information and spectra for calcium-rich phosphate minerals other than apatite-group minerals are presented in Table 2 ▶ and Fig. 2 ▶, respectively. Non-apatite calcium phosphates displayed a primary absorption peak between 2153.0 and 2154.0 eV. Non-apatitic calcium phosphates also displayed a shoulder on the main peak, much like the apatite-group minerals (Fig. 2 ▶). Similar shoulders are observed for other non-apatitic calcium phosphates like brushite in other studies (Shober *et al.*, 2006 ▶). Although this shoulder is most pronounced in calcium phosphates, a small post-peak shoulder has also been reported in laboratory-grade potassium phosphate salt (Brandes *et al.*, 2007 ▶). Distinguishing between calcium minerals and potassium phosphates is possible through several differences in secondary and tertiary peak positions (Brandes *et al.*, 2007 ▶). Notably, the shoulders for the non-apatite calcium phosphate minerals presented in Fig. 2 ▶ are not nearly as distinct as those observed for apatite-group minerals (Fig. 1 ▶). These observations suggest that the presence and strength of this shoulder are related to the presence and abundance of calcium in the mineral structure. Monetite has been suggested as an important precursor phase for the growth of apatite minerals in natural systems (Van Cappellen & Berner, 1988 ▶). Consistent with this suggestion, the monetite spectrum in Fig. 2 ▶ is somewhat similar to that of poorly crystalline apatite in Fig. 1 ▶, except that the secondary peaks of the monetite are found at relatively lower energies.

### 3.3. Aluminium phosphate minerals

**Figure 3:**
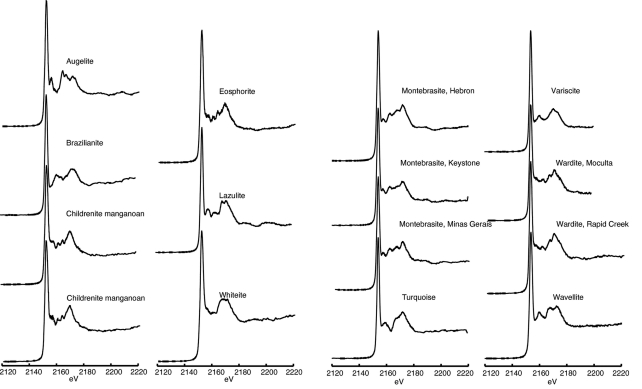
Phosphorus XANES spectra for aluminium phosphate minerals listed in Table 3 ▶.

Information and spectra for aluminium-rich phosphate minerals are presented in Table 3 ▶ and Fig. 3 ▶, respectively. Characterization of aluminium-phosphate-containing phases in soils has been the focus of many studies owing to the role of these minerals in controlling the phosphorus availability to plants (Hesterberg *et al.*, 1999 ▶; Shober *et al.*, 2006 ▶; Khare *et al.*, 2005 ▶). Soluble salts of aluminium have been added to materials such as poultry litter in order to sequester phosphorus *via* formation of relatively insoluble aluminium phosphate minerals (Seiter *et al.*, 2008 ▶; Peak *et al.*, 2002 ▶). A narrow *K*-edge peak (∼0.9 eV full width at half-maximum) characterizes the spectra for aluminium phosphate minerals. This mineral group lacks the shoulder seen in the calcium phosphate mineral spectra. A distinctive feature common to all aluminium phosphate mineral spectra is the secondary peak centered at approximately 2175 eV. The positions of other higher-energy peaks are variable, likely reflecting differences in structure and composition between aluminium phosphate mineral phases. The similarity of the three montebrasite samples is notable given that the idealized structural formula allows for compositional variation in terms of lithium, sodium, hydroxyl and fluorine content. Spectra of the two childrenite manganoan samples, which exhibited distinctly different colors in hand specimen, are also identical. The absence of spectral variation in the mineral groups above is similar to the case for apatite-group minerals which have variations in composition, yet also show little variation in XANES spectra (Fig. 1 ▶, Table 1 ▶). These trends suggest that within a specific mineral structure small compositional variations typical of natural samples do not result in obvious differences in XANES spectra. Conversely, minerals such as the hydrous phosphates of aluminium, which have approximately similar composition but different crystal structures, exhibit notably different post-edge features in their XANES spectra (*e.g.* augelite, variscite and wavellite).

### 3.4. Iron and manganese phosphate minerals

**Figure 4:**
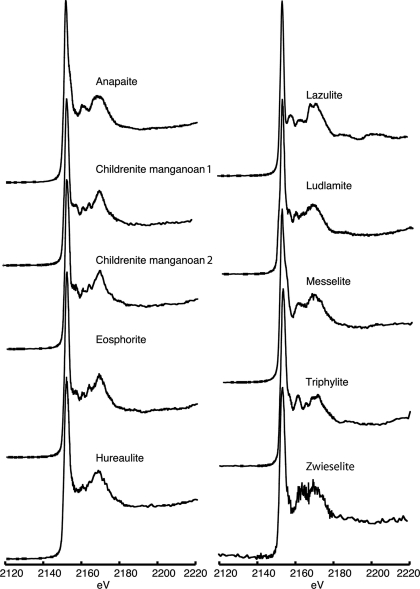
Phosphorus XANES spectra for phosphate minerals containing reduced iron or manganese listed in Table 4 ▶.

**Figure 5:**
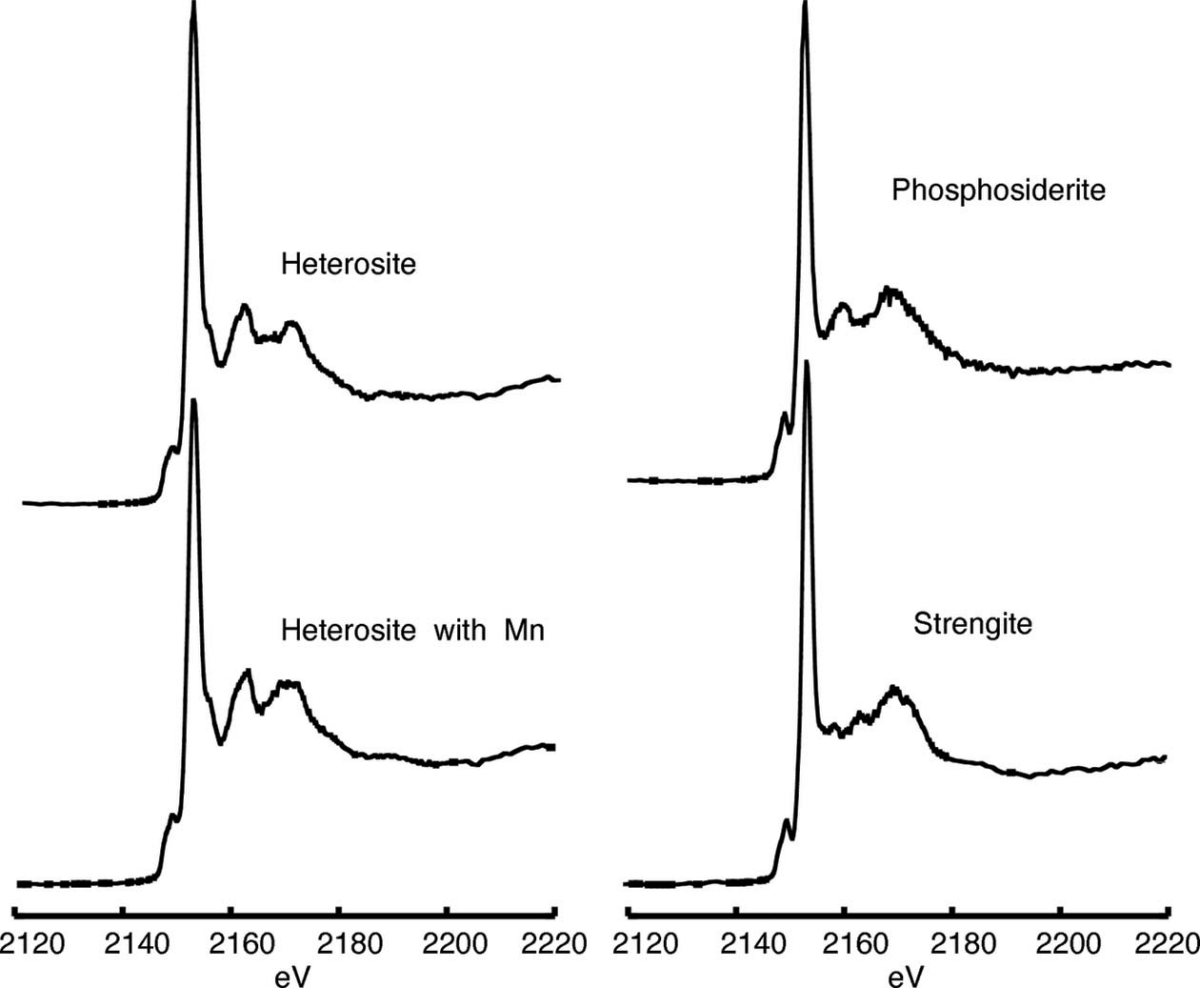
Phosphorus XANES spectra for phosphate minerals containing oxidized iron or manganese listed in Table 5 ▶.

A shift from oxidizing to reducing conditions in aquatic systems and soils commonly results in the mobilization of adsorbed phosphorus from solids to the dissolved phase. Explanations regarding the sensitivity of phosphorus burial and preservation to the redox potential of the surrounding environment have often invoked the cycling of adsorbed phosphorus associated with reducible manganese and iron oxide phases (Froelich *et al.*, 1982 ▶; Sundby *et al.*, 1992 ▶; Ingall & Jahnke, 1994 ▶; Ingall *et al.*, 2005 ▶; McManus *et al.*, 1997 ▶). Iron and manganese typically substitute for one another in many phosphate minerals because similarity in charge and ionic radii allow these elements to occupy the same site in a mineral structure. Because of this trend, information and spectra for phosphate minerals containing iron and manganese are grouped together. Reduced iron- and manganese-containing phosphate minerals are presented in Table 4 ▶ and Fig. 4 ▶, and oxidized iron- and manganese-containing phosphate minerals are presented in Table 5 ▶ and Fig. 5 ▶. As seen in Table 4 ▶, phosphate minerals containing reduced iron and manganese typically contain other elements such as calcium, aluminium, magnesium, lithium and fluorine in addition to hydrogen and oxygen (either as hydroxyl or water). Reduced iron- and manganese-containing phosphate minerals exhibit a large degree of diversity in post-edge features, which is consistent with the compositional and structural diversity of these minerals. Childrenite and eosphorite have very similar spectra (Fig. 4 ▶), which is expected because these minerals share the same structure and differ only by the extent to which iron substitutes for manganese.

Phosphate minerals containing oxidized iron and manganese typically form as weathering or other alteration products of parent minerals. Often these alteration products are intimately mixed with parent materials, making the acquisition of standards with appropriate phase purity difficult. A distinctive feature of the oxidized iron and manganese phosphate minerals is the unique pre-edge feature present at 2150.1 eV. Peak fitting of the phosphosiderite spectrum indicated the presence of a second pre-edge peak at 2149.1 eV as well. Other studies have indicated that pre-edge features are present in other oxidized iron phosphates (Pratesi *et al.*, 2003 ▶; Hesterberg *et al.*, 1999 ▶; Khare *et al.*, 2004 ▶, 2005 ▶) or in reduced iron phosphate minerals such as vivianite that have been exposed to oxidizing conditions for extended time periods (Brandes *et al.*, 2007 ▶). This feature, then, is useful as an indicator of the oxidation state of iron associated with phosphate, even in samples with other iron phases. Similarly, well defined pre-edge features have been observed in chromium and cobalt phosphate materials (Okude *et al.*, 1999 ▶), but such phases are extremely rare in natural systems and can be distinguished from iron and manganese phosphate minerals using higher-energy secondary peaks. Both heterosite samples exhibit a small shoulder at approximately 2157 eV on the *K*-edge peak (Fig. 5 ▶). Although a shoulder in this region is one characteristic typical of calcium phosphate minerals, the combination of the pre-edge feature and the higher-energy features can be used to distinguish heterosite from calcium phosphate minerals (Figs. 1 ▶ and 5 ▶).

### 3.5. Magnesium, copper, zinc and rare-earth phosphate minerals

**Figure 6:**
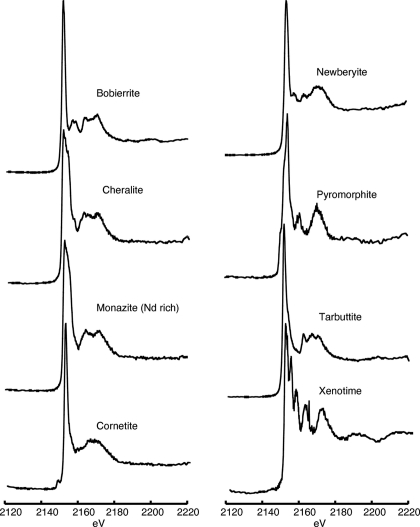
Phosphorus XANES spectra for magnesium, copper, zinc and rare-earth phosphate minerals listed in Table 6 ▶.

Information and spectra for magnesium, copper, zinc and rare-earth phosphate minerals are presented in Table 6 ▶ and Fig. 6 ▶, respectively. In sediments the presence of finely dispersed phosphate minerals that contain rare-earth elements has been suggested as a significant phosphorus removal mechanism in ancient marine systems (Rasmussen, 1996 ▶). Other minerals presented in this group such as pyromorphite, cornetite and tarbuttite are often associated with ore bodies of lead, copper and zinc, respectively. The minerals of these groups can be clearly distinguished by their post-edge spectral features.

Phosphate minerals containing rare-earth elements (*e.g.* cheralite, monazite and xenotime) have quite distinctive spectra. Xenotime has five sharp resonance peaks in the post-edge region. These resonances result from the interactions between the electronic structure of phosphate and the outer orbitals of group 3 elements (Okude *et al.*, 1999 ▶). Cheralite and monazite have noticeably broad peaks at the absorption edge that likely result from a multiplicity of resonances resulting from the mixture of elements found in these minerals. Cornetite has a distinctive pre-edge feature (two peaks at 2149.5 eV and 2151.6 eV), and pyromorphite has two small pre-edge shoulders that are difficult to discern at the scale of Fig. 6 ▶ (see online supplementary material).

### 3.6. Uranium phosphate minerals

**Figure 7:**
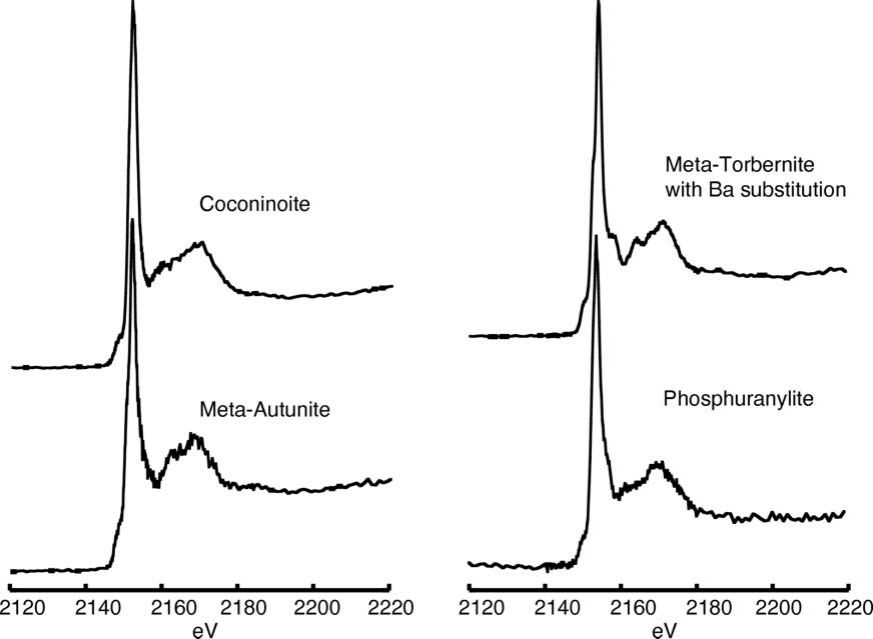
Phosphorus XANES spectra for uranium phosphate minerals listed in Table 7 ▶.

Information and spectra for uranium phosphate minerals are presented in Table 7 ▶ and Fig. 7 ▶, respectively. Uranium phosphate minerals often occur as coatings on rock surfaces or dispersed between grains of sediments (Burns & Finch, 1999 ▶). These minerals also tend to exhibit a wide range of composition with extensive substitution of the major cation being typical (Burns & Finch, 1999 ▶). Thus, they are typically difficult to identify and characterize in natural environments. In addition to the study of naturally occurring uranium phosphates, anthropogenically enhanced microbially mediated precipitation of uranium phosphate minerals has been proposed as a mechanism for uranium sequestration in contaminated systems (Beazley *et al.*, 2009 ▶).

The mole percent of uranium is below 10% for all minerals listed in Table 7 ▶. All the uranium minerals exhibit a common pre-edge feature just to the low-energy side of the main peak, a doublet peak at 2151 and 2152 eV (Fig. 7 ▶). The higher-energy spectral features observed in Fig. 7 ▶ are not dominated by any particular peaks, which are often observed in minerals with high mole percentages of one particular cation (*e.g.* apatite-group minerals, Fig. 1 ▶). A rough similarity of the spectra likely results from the incorporation of uranium in these mineral structures as the UO_2_^2−^ anion, which limits electron interactions with phosphorus and other elements in the mineral structure to more long-range effects. The two uranium minerals that contain calcium, meta-autunite (2.3 mol% calcium) and phosphuranylite (1.2 mol% calcium), have a weak shoulder on the high-energy side of the primary peak. This feature was clearly developed in the calcium phosphate minerals discussed above, but was, as expected, less developed in those minerals with much lower calcium contents.

## 4. Conclusions

Examination of 44 phosphorus mineral standards indicates that pre-edge and post-edge energy features can be used to identify specific minerals or differentiate between different phosphate mineral groups. Variations in *K*-edge and primary peak positions are relatively small across phosphorus mineral phases and therefore require careful attention to energy calibration and stability to be useful as diagnostic features. In addition to mineral composition, mineral structure greatly influences the position of post-edge spectral features. In particular, greater consistency was found in the XANES spectra from minerals sharing similar structure rather than similar composition. This reflects the presence of structure-dependent low-*k* EXAFS (extended X-ray absorption fine structure) oscillations in the near-edge spectral region. Utilizing all these features, XANES provides a powerful tool for investigating the composition and cycling of phosphorus in natural and artificial systems. In particular, for many natural systems phosphorus minerals occur as submicrometer and micrometer-sized particles in heterogeneous and complex matrices, which preclude direct characterization by simple, chemical, optical or bulk spectroscopic techniques. In such systems, focused-beam X-ray spectroscopic techniques provide excellent avenues to investigate phosphorus chemistry.

## Supplementary Material

Supplementary material file. DOI: 10.1107/S0909049510045322/hi5614sup1.xls

## Figures and Tables

**Table 1:** Names, localities and formulas for apatite-group minerals

Mineral	Locality	Ideal formula	Wt% C
Apatite (poorly crystalline)	Mono Lake, Mono County, CA, USA	Ca_5_(PO_4_)_3_(OH,F)	
Carbonate apatite	Brewster, FL, USA	Ca_5_(PO_4_,CO_3_)_3_(OH,F)	0.81
Carbonate fluorapatite	Stoke Climstand, East Cornwall, England	Ca_5_(PO_4_,CO_3_)_3_(F)	0.26
Carbonate fluorapatite	St Just, Cornwall, England	Ca_5_(PO_4_,CO_3_)_3_(F)	0.08
Carbonate hydroxylapatite fluorian	Snarnum, Norway	Ca_5_(PO_4_,CO_3_)_3_(OH,F)	0.13
Fluorapatite	Durango, Mexico	Ca_5_(PO_4_)_3_F	
Fluorapatite	Ipira, Minas Gerais, Brazil	Ca_5_(PO_4_)_3_F	
Fluorapatite	Renfrew County, Ontario, Canada	Ca_5_(PO_4_)_3_F	
Fluorapatite	Beartooth Mountains, WY, USA	Ca_5_(PO_4_)_3_F	
Fluorapatite	Conda, ID, USA	Ca_5_(PO_4_)_3_F	
Fluorapatite	Conselheiro Pena, Minas Gerais, Brazil	Ca_5_(PO_4_)_3_F	
Hydroxylapatite chlorian	Bamle, Telemark, Norway	Ca_5_(PO_4_)_3_(OH,Cl)	

**Table 2:** Names, localities and formulas for calcium phosphate minerals other than apatite group members

Mineral	Locality	Ideal formula	Ideal mol% Ca
Anapaite	Kerch Pennisula, Crimea, Ukraine	Ca_2_Fe(PO_4_)_2_·4H_2_O	8
Herderite	Linopolis, Minas Gerais, Brazil	CaBe(PO_4_)F	12.5
Messelite	Bessemer City, NC, USA	Ca_2_(Mn,Fe^2+^)(PO_4_)_2_·2H_2_O	10.5
Monetite	Synthetic (Alfa Chemical 40232)	CaHPO_4_	14.3
Scholzite	Blinman, SA, Australia	CaZn_2_(PO_4_)_2_·2H_2_O	5.3
Whiteite	Rapid Creek, Yukon Territory, Canada	(Ca,Fe,Mg)_2_Al_2_(PO_4_)_4_(OH)_2_·8H_2_O	3.8

**Table 3:** Names, localities and formulas for aluminium phosphate minerals

Mineral	Locality	Ideal formula	Ideal mol% Al
Augelite	Rapid Creek, Yukon, Canada	Al_2_(PO_4_)(OH)_3_	15.4
Brazilianite	Minas Gerais, Brazil	NaAl_3_(PO_4_)_2_(OH)_4_	13.6
Childrenite manganoan 1	Minas Gerais, Brazil	(Mn,Fe)Al(PO_4_)(OH)_2_·H_2_O	7.1
Childrenite manganoan 2	Minas Gerais, Brazil	(Mn,Fe)Al(PO_4_)(OH)_2_·H_2_O	7.1
Eosphorite	Rio Grande de Norte, Brazil	MnAl(PO_4_)(OH)_2_·H_2_O	7.1
Lazulite	Rapid Creek, Yukon, Canada	(Mg,Fe)Al_2_(PO_4_)_2_(OH)_2_	11.7
Montebrasite	Hebron, ME, USA	(Li,Na)Al(PO_4_)(OH,F)	12.5
Montebrasite	Keystone, SD, USA	(Li,Na)Al(PO_4_)(OH,F)	12.5
Montebrasite	Minas Gerais, Brazil	(Li,Na)Al(PO_4_)(OH,F)	12.5
Turquoise	Bisbee, AZ, USA	CuAl_6_(PO_4_)_4_(OH)_8_·5H_2_O	12.2
Variscite	Avant, AR, USA	AlPO_4_·2H_2_O	9.1
Wardite	Moculta, SA, Australia	NaAl_3_(PO_4_)_2_(OH)_4_·2(H_2_O)	10.7
Wardite	Rapid Creek, Yukon, Canada	NaAl_3_(PO_4_)_2_(OH)_4_·2(H_2_O)	10.7
Wavellite	Mount Ida, AR, USA	Al_3_(PO_4_)_2_(OH,F)_3_·5(H_2_O)	9.7
Whiteite	Rapid Creek, Yukon, Canada	(Ca,Mn,Mg)_2_Al_2_(PO_4_)_4_(OH)_2_·8(H_2_O)	3.8

**Table 4:** Names, localities and formulas for phosphate minerals containing reduced iron and manganese [Fe(II), Mn(II)]

Mineral	Locality	Ideal formula	Ideal mol% Fe or Mn
Anapaite	Kerch Pennisula, Crimea, Ukraine	Ca_2_Fe(PO_4_)_2_·4H_2_O	4
Childrenite manganoan 1	Minas Gerais, Brazil	(Mn,Fe)Al(PO_4_)(OH)_2_·H_2_O	7.1
Childrenite manganoan 2	Minas Gerais, Brazil	(Mn,Fe)Al(PO_4_)(OH)_2_·H_2_O	7.1
Eosphorite	Rio Grande de Norte, Brazil	MnAl(PO_4_)(OH)_2_·H_2_O	7.1
Hureaulite	Minas Gerais, Brazil	Mn_5_(PO_3_OH)_2_(PO_4_)_2_·4H_2_O	12.8
Lazulite	Rapid Creek, Yukon, Canada	(Mg,Fe)Al_2_(PO_4_)_2_(OH)_2_	5.9
Ludlamite	Lemhi County, ID, USA	(Fe,Mg,Mn)_3_(PO_4_)_2_·4H_2_O	12
Messelite	Bessemer City, NC, USA	Ca_2_(Mn,Fe)(PO_4_)_2_·2H_2_O	5.3
Triphylite	Center Stafford, NH, USA	Li(Fe, Mn)PO_4_	14.3
Zwieselite	Fremont County, CO, USA	(Fe,Mn)_2_(PO_4_)F	22.2

**Table 5:** Names, localities and formulas for phosphate minerals containing oxidized iron and manganese [Fe(III), Mn(III)]

Mineral	Locality	Ideal formula	Ideal mol% Fe or Mn
Heterosite	Palermo Mine, NH, USA	Fe(PO_4_)	16.7
Heterosite with Mn	Karibib District, Erongo Region, Namibia	(Fe,Mn)(PO_4_)	16.7
Phosphosiderite	Coosa County, AL, USA	FePO_4_·2H_2_O (monoclinic)	8.3
Strengite	Indian Mountain, AL, USA	FePO_4_·2H_2_O (orthorhombic)	8.3

**Table 6:** Names, localities and formulas for magnesium, copper, lead, zinc and rare-earth phosphate minerals

Mineral	Locality	Ideal formula	Ideal mol% metal
Bobierrite	Kovdov, Kola Peninsula, Russia	Mg_3_(PO_4_)_2_·8H_2_O	8.1
Cheralite	Yampa, CO, USA	(Ln,Th,Ca,U)(PO_4_,SiO_4_)	16.7
Cornetite	Lumbubashi, Shaba, Republic of Congo	Cu_3_(PO_4_)(OH)_3_	21.4
Monazite (Nd-rich)	Rio Arriba County, NM, USA	(Nd,Ce,La,Th,Y)PO_4_	16.7
Newberyite	Skipton Bat Caves, Victoria, Australia	Mg(HPO_4_)·3H_2_O	6.3
Pyromorphite	Yangshu, Guangxi Provence, China	Pb_5_(PO_4_)_3_Cl	23.8
Tarbuttite	Broken Hill Mine, Zambia	Zn_2_(PO_4_)(OH)	22.2
Xenotime	Beryl Hill, Australia	YPO_4_	16.7

**Table 7:** Names, localities and formulas for uranium phosphate minerals

Mineral	Locality	Ideal formula	Ideal mol% U
Coconinoite	San Juan County, UT, USA	Fe_2_Al_2_(UO_2_)_2_(PO_4_)_4_(SO_4_)(OH)_2_·18H_2_O	2.1
Meta-autunite	Spokane, WA, USA	Ca(UO_2_)_2_(PO_4_)_2_·10H_2_O	4.7
Meta-torbernite with Ba substitution	Spruce Pine Pegmatite District, NC, USA	(Cu,Ba)(UO_2_)_2_(PO_4_)_2_·8H_2_O	4.9
Phosphuranylite	Grafton County, NH, USA	KCa(H_3_O)_3_(UO_2_)_7_(PO_4_)_4_O_4_·8H_2_O	8.4

## Footnotes

1 Supplementary data for this paper are available from the IUCr electronic archives (Reference: HI5614). Services for accessing these data are described at the back of the journal.
